# Synthesis, crystal structure and Hirshfeld analysis of *N*-ethyl-2-{3-methyl-2-[(2*Z*)-pent-2-en-1-yl]cyclo­pent-2-en-1-yl­idene}hydrazinecarbo­thio­amide

**DOI:** 10.1107/S2056989024002913

**Published:** 2024-04-09

**Authors:** Adriano Bof de Oliveira, Johannes Beck, Jörg Daniels

**Affiliations:** a Departamento de Química, Universidade Federal de Sergipe, Av. Marcelo Deda Chagas s/n, Campus Universitário, 49107-230 São Cristóvão-SE, Brazil; b Institut für Anorganische Chemie, Rheinische Friedrich-Wilhelms-Universität Bonn, Gerhard-Domagk-Strasse 1, D-53121 Bonn, Germany; Universität Greifswald, Germany

**Keywords:** thio­semicarbazone, jasmone, jasmone 4-ethyl­thio­semicarbazone, H-bonded ribbon, crystal structure, Hirshfeld analysis

## Abstract

The title compound (C_14_H_23_N_3_S, common name: *cis*-jasmone 4-ethyl­thio­semicarbazone) was synthesized by the equimolar reaction of *cis*-jasmone and 4-ethyl­thio­semicarbazide in ethanol facilitated by acid catalysis. There is one crystallographically independent mol­ecule in the asymmetric unit, which shows disorder of the terminal ethyl group of the jasmone carbon chain [site-occupancy ratio = 0.911 (5):0.089 (5)]. The thio­semicarbazone entity [N—N—C(=S)—N] is approximately planar, with the maximum deviation of the mean plane through the N/N/C/S/N atoms being 0.0331 (8) Å, while the maximum deviation of the mean plane through the five-membered ring of the jasmone fragment amounts to −0.0337 (8) Å. The dihedral angle between the two planes is 4.98 (7)°. The mol­ecule is not planar due to this structural feature and the *sp* ^3^-hybridized atoms of the jasmone carbon chain. Additionally, one H⋯N intra­molecular inter­action is observed, with graph-set motif *S*(5). In the crystal, the mol­ecules are connected through pairs of H⋯S inter­actions with *R*_2_^2^(8) and *R*_2_^1^(7) graph-set motifs into centrosymmetric dimers. The dimers are further connected by H⋯N inter­actions with graph-set motif *R*_2_^2^(12), which are related by an inversion centre, forming a mono-periodic hydrogen-bonded ribbon parallel to the *b*-axis. The crystal structure and the supra­molecular assembly of the title compound are compared with four known *cis*-jasmone thio­semicarbazone derivatives (two crystalline modifications of the non-substituted form, the 4-methyl and the 4-phenyl derivatives). A Hirshfeld surface analysis indicates that the major contributions for the crystal cohesion are from H⋯H (70.7%), H⋯S/S⋯H (13.5%), H⋯C/C⋯H (8.8%), and H⋯N/N⋯H (6.6%) inter­faces (only the disordered atoms with the highest s.o.f. were considered for the evaluation).

## Abstract 2

The synthesis, crystal structure and Hirshfeld analysis of *cis*-jasmone 4-ethyl­thio­semicarbazone are reported. The crystallographically independent mol­ecule shows disorder over the jasmone carbon chain and, in the crystal, the mol­ecules are linked by H⋯S and H⋯N inter­actions into mono-periodic hydrogen-bonded ribbons parallel to the *b*-axis.

## 1. Chemical context

Thio­semicarbazones [*R*_1_ *R*_2_C=N—N(H)C(=S)N*R*_3_ *R*_4_] can be readily prepared through a well-known condensation reaction between a ketone or an aldehyde (*R*_1_ *R*_2_C=O) and a thio­semicarbazide derivative [H_2_N—N(H)C(=S)N*R*_3_ *R*_4_] (Freund & Schander, 1902 ▸). Due to the structural diversity of the educts, a huge number of thio­semicarbazone derivatives (**TSC**) can be synthesized for numerous applications across a wide range of scientific disciplines, such as coordination chemistry, medicinal chemistry and materials science. These three main approaches are inter­connected, as demonstrated by Farias *et al.* (2021 ▸) in a report concerning the synthesis, *in vitro* and *in silico* evaluations of the anti­tumor activities of two thio­semicarbazone Ni^II^ complexes, which were considered materials with biological properties. The coordination chemistry of thio­semicarbazone derivatives was addressed in a review by Lobana *et al.* (2009 ▸), illustrating the chemical bonding of **TSC**s with metal centres of different Lewis acidity, the coordination modes and geometries, and some biological and analytical applications.

Several thio­semicarbazone derivatives have biological properties either as metal complexes or as non-coordinated mol­ecules. For examples of thio­semicarbazone complexes with biological activity, see: Gupta *et al.* (2022 ▸); Khan *et al.* (2022 ▸); Monsur Showkot Hossain *et al.* (2023 ▸); Parrilha *et al.* (2022 ▸), which covers compounds for chemotherapy and medical diagnostic imaging combined, also referred to as theranostics, and Singh *et al.* (2023 ▸). For a review of **TSC** complexes in the inhibition of topoisomerases, which are biological targets of prime importance in cancer research, see: Jiang *et al.* (2023 ▸). For examples of the biological activity of non-coordinated thio­semicarbazone derivatives, see: Fatondji *et al.* (2013 ▸), which shows a small chemical library with 35 derivatives with trypanocidal activity against the *Trypanosoma brucei brucei* parasite, and for a review on tyrosinase inhibitory activity, which is another important biological target, see: Hałdys & Latajka (2019 ▸). The non-coordinated **TSC**s are also mentioned in a review on tyrosinase inhibition by Zolghadri *et al.* (2019 ▸). In addition, thio­semicarbazone derivatives have been studied for the treatment of Parkinson’s disease (Mathew *et al.*, 2021 ▸), microbial growth inhibition (D’Agostino *et al.*, 2022 ▸), anti-inflammatory pathologies (Kanso *et al.*, 2021 ▸) and anti­fungal activity (Bajaj *et al.*, 2021 ▸). Specifically, in the context of this work, the parent *cis*-jasmone thio­semicarbazone derivative has shown fungistatic biological activity as a free mol­ecule (Jamiołkowska *et al.*, 2022 ▸) and also as a Cu^II^ complex (Orsoni *et al.*, 2020 ▸).

In materials science, thio­semicarbazone complexes are employed as single-source educts for the synthesis of nanostructured materials, *e.g.*, CdS nanocrystals (Masikane *et al.*, 2019 ▸) and nanostructured CuFeS_2_, which is being used as an electrode material for supercapacitors (Ansari *et al.*, 2022 ▸). Non-coordinated thio­semicarbazones have been used to functionalize metal–organic frameworks (MOFs), such as zeolitic imidazolate frameworks (mainly, ZIF-8), for the removal of Hg^II^ from aqueous solutions at room temperature and neutral pH (Jaafar *et al.*, 2021 ▸). **TSC** derivatives have also been studied as corrosion inhibitors for metals and alloys. For the respective theoretical approach, see: Silva & Martínez-Huitle (2021 ▸). Additionally, thio­semicarbazone derivatives have turned out to be useful in several fields of analytical chemistry, including calorimetry, fluorimetry and electrochemical sensors, *e.g.*, in the detection of anions and metallic cations (Özbek & Berkel, 2023 ▸). 
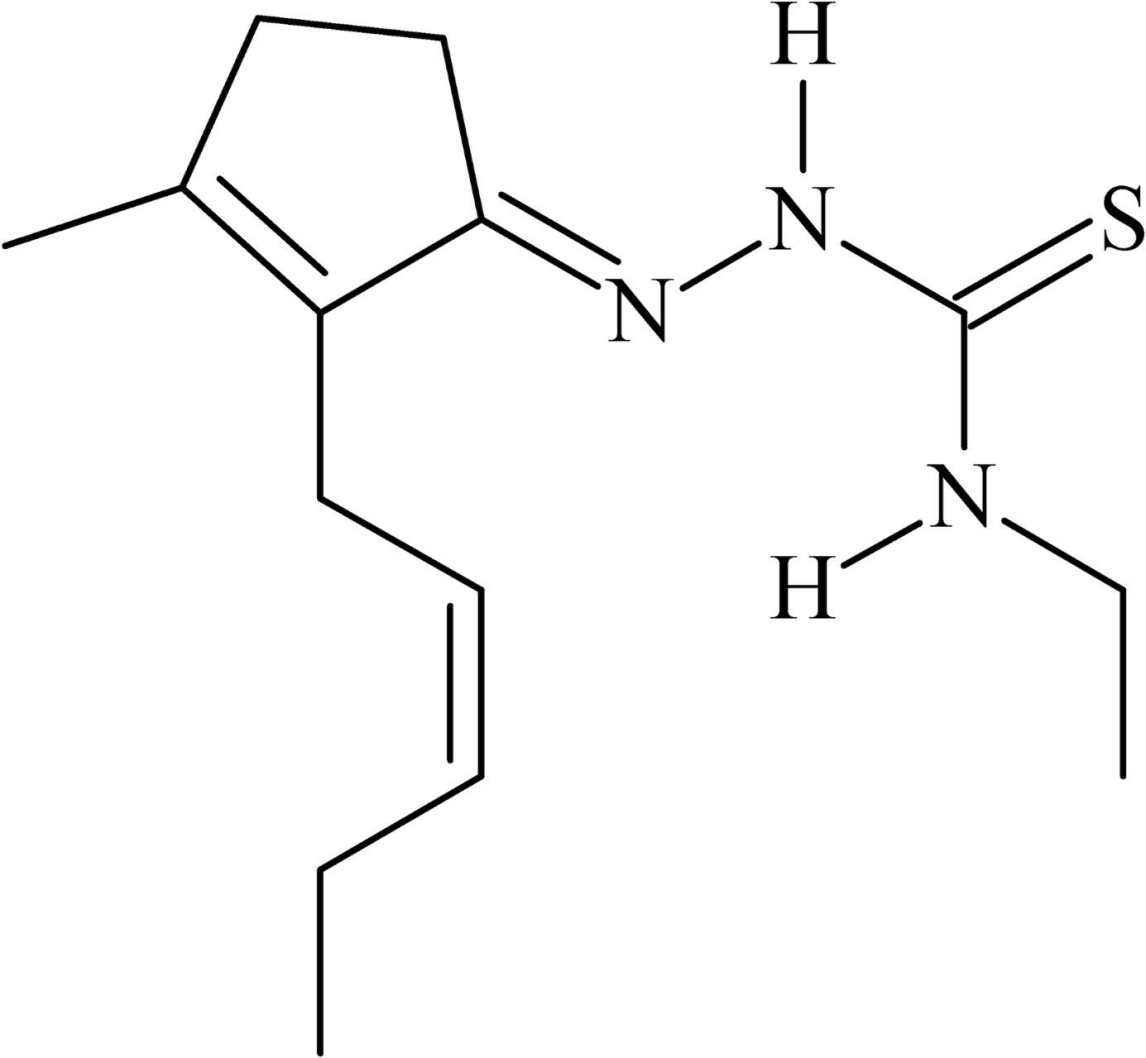


In this context and as a contribution to the **TSC** chemistry, we report here the synthesis, crystal structure and Hirshfeld analysis of *cis*-jasmone 4-ethyl­thio­semicarbazone.

## 2. Structural commentary

**Figure 1:**
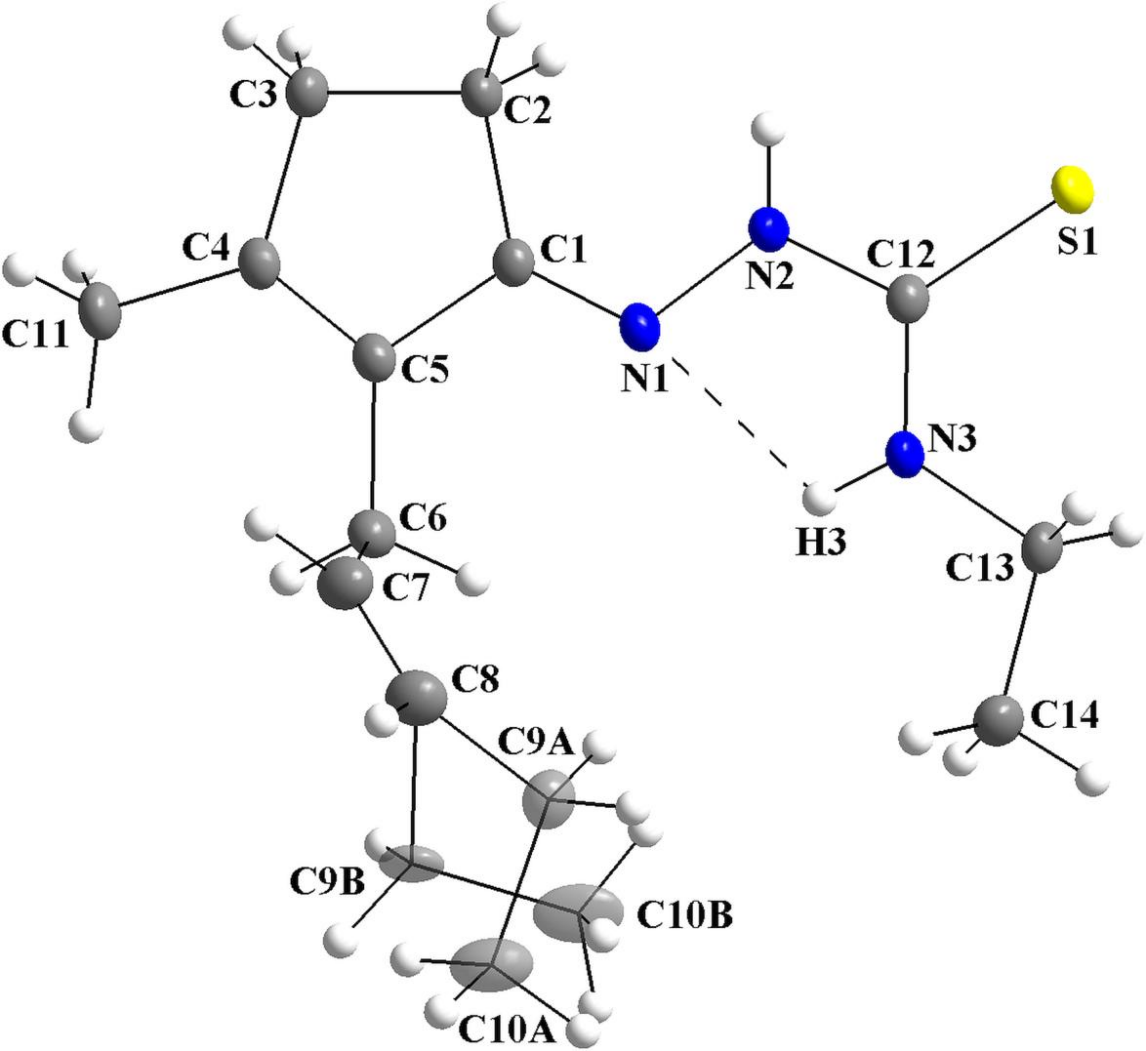
The mol­ecular structure of the title compound, showing the atom labelling and displacement ellipsoids drawn at the 40% probability level. Disordered atoms are drawn with 40% transparency and labelled C9*A*/C10*A* [s.o.f. = 0.911 (5)] and C9*B*/C10*B* [s.o.f. = 0.089 (5)]. For the N3—H3⋯N1 intra­molecular inter­action, a ring with graph-set motif *S*(5) is observed.

For the title compound, *cis*-jasmone 4-ethyl­thio­semicarbazone (**JETSC**), the asymmetric unit consists of one mol­ecule with all atoms in general positions, which shows disorder over the jasmone carbon chain [s.o.f. = 0.911 (5):0.089 (5)]. The disordered atoms with higher s.o.f. are *A*-labelled and the atoms with lower s.o.f. are *B*-labelled (Fig. 1 ▸). The thio­semicarbazone entity is approximately planar, with the maximum deviation of the mean plane through the N1/N2/C12/S1 atoms being 0.0331 (8) Å for N2 (r.m.s.d. = 0.0215 Å). For the five-membered ring of the jasmone fragment, the maximum deviation of the mean plane through the selected atoms amounts to −0.0337 (8) Å for C2 (r.m.s.d. = 0.0256 Å) and the dihedral angle between the two planes is 4.98 (7)°. The mol­ecule is not planar due to this angle and to the *sp* ^3^-hybridized atoms of the jasmone carbon chain, with the torsion angles for the C5—C6—C7—C8, C7—C8—C9*A*—C10*A* and C7—C8— C9*B*—C10*B* fragments being −138.74 (16), −106.5 (2) and 132.7 (10)°. Finally, an intra­molecular hydrogen-bond inter­action is observed, N3—H3⋯N1, which forms a ring of graph-set motif *S*(5) (Fig. 1 ▸ and Table 1 ▸). For a review addressing hydrogen bonding in the solid state, see: Steiner (2002 ▸).

## 3. Supra­molecular features

**Figure 2:**
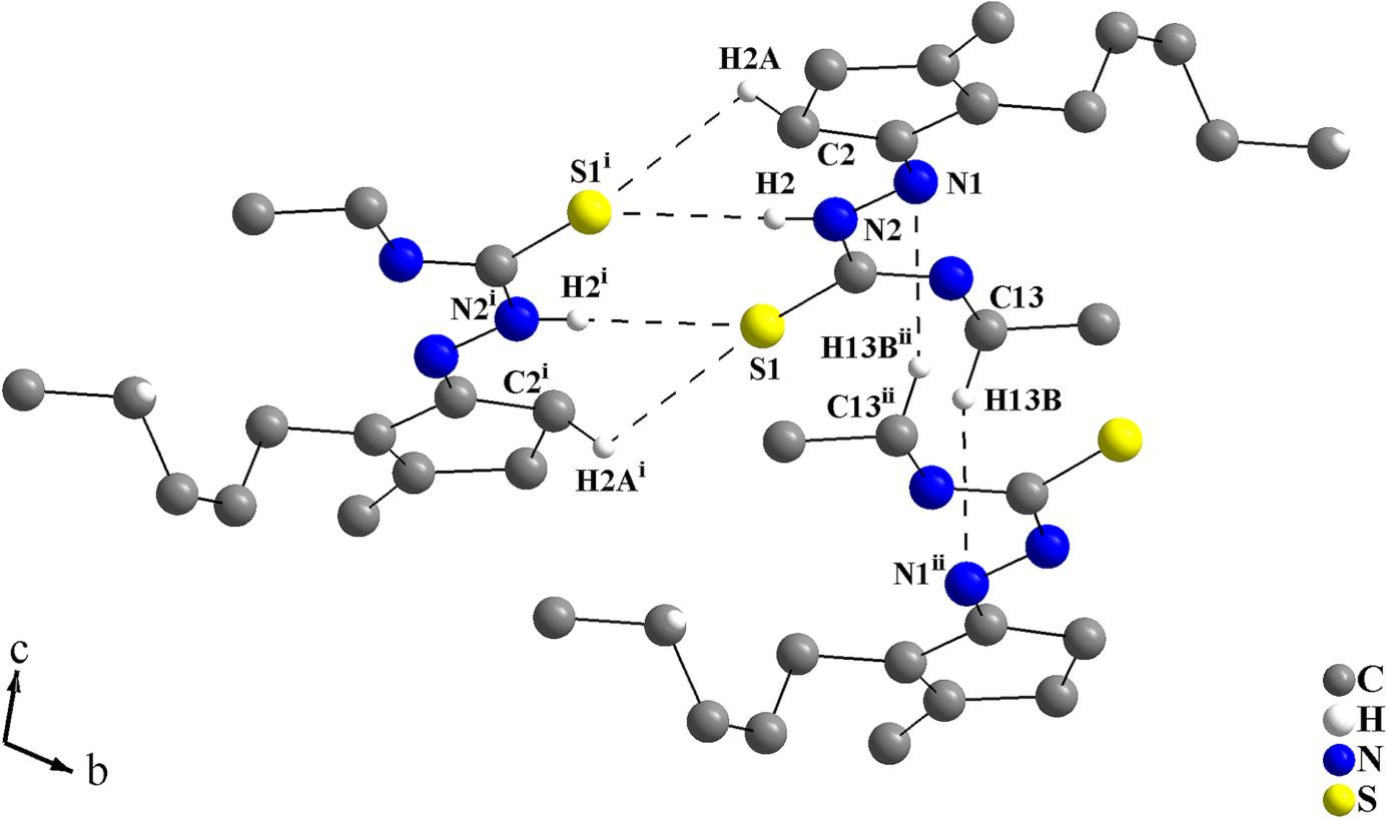
Crystal structure section of the title compound showing the inter­molecular hydrogen-bonding inter­actions as dashed lines. The mol­ecules are linked *via* pairs of C2—H2*A*⋯S1^i^, N—H2⋯S1^i^ and C13—H13*B*—N1^ii^ inter­actions with graph-set motifs 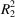 (8), 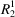 (7) and 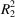 (12). [Symmetry codes: (i) −*x* + 1, −*y*, −*z* + 1; (ii) −*x* + 1, −*y* + 1, −*z* + 1.]

**Figure 3:**
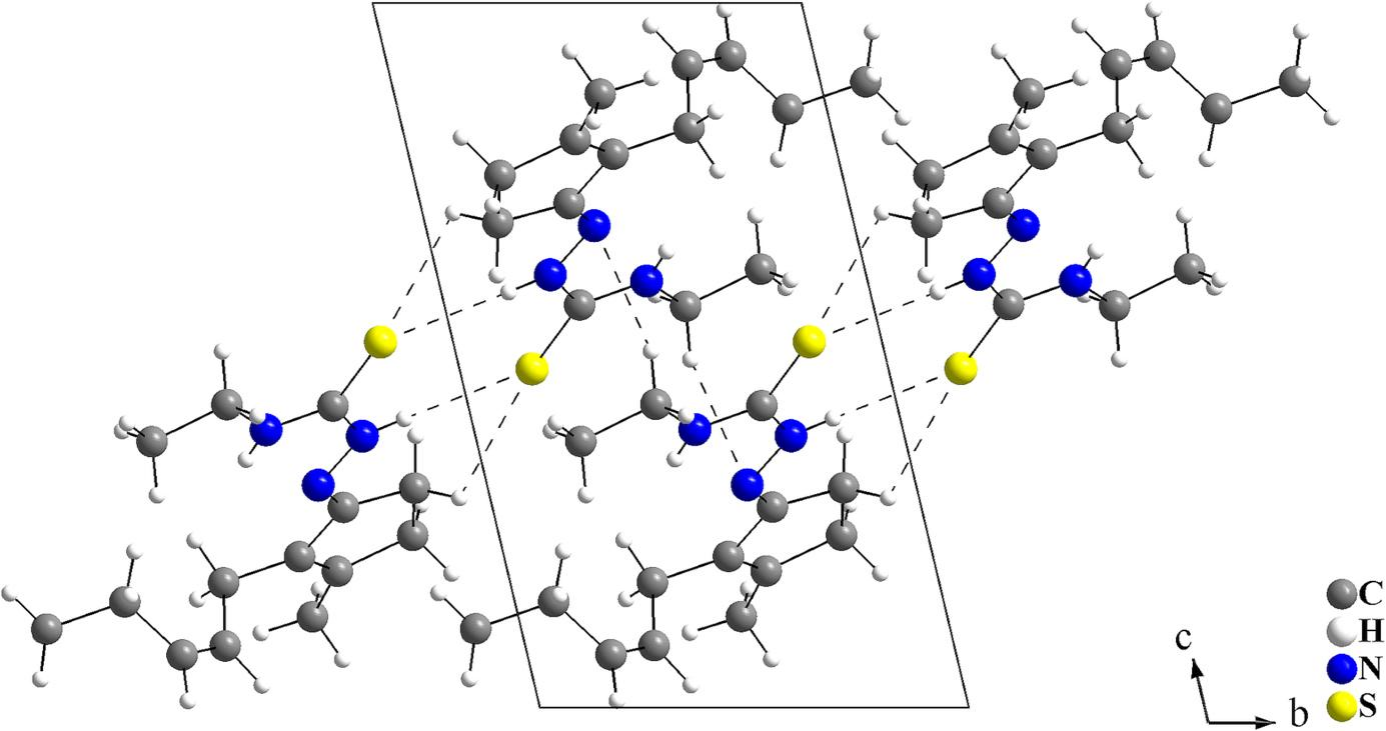
Crystal structure section of the title compound, showing the inter­molecular hydrogen-bonded inter­actions as dashed lines. Disorder is not shown for clarity. The mol­ecules are linked into mono-periodic hydrogen-bonded ribbons parallel to the *b*-axis *via* pairs of N—H⋯S, C—H⋯S and C—H⋯N inter­actions with graph-set motifs 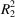 (8), 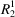 (7) and 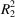 (12).

In the crystal, the mol­ecules are connected through H⋯S and H⋯N inter­actions, forming rings of graph-set motifs 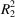 (8), 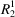 (7) and 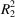 (12) for the C_2_H_2_N_2_S_2_, C_2_H_2_N_2_S and C_4_H_2_N_6_ entities, respectively (Fig. 2 ▸). The S1 and N1 atoms act as double hydrogen-bond acceptors, where the N1 atoms play an important role in the supra­molecular arrangement of the mol­ecules. Firstly, the mol­ecules are connected into centrosymmetric dimers through C2—H2*A*⋯S1^i^ and N2—H2⋯S1^i^ inter­molecular inter­actions [symmetry code: (i) −*x* + 1, −*y*, −*z* + 1], as has also been observed in other *cis*-jasmone thio­semicarbazone derivatives (Oliveira *et al.*, 2023*a* ▸, 2024 ▸). These centrosymmetric dimers, in which rings of graph-set motif 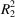 (8) and 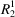 (7) are present, have their centres of gravity located in the centre of the *ac* planes. In addition, the dimers are further connected by C13—H13*B*⋯N1^ii^ inter­molecular inter­actions [symmetry code: (ii) −*x* + 1, −*y* + 1, −*z* + 1], where rings of graph-set motif 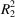 (12) are observed (Fig. 2 ▸, Table 1 ▸). The centre of gravity of the centrosymmetric C_4_H_2_N_6_ ring lies at an inversion centre of the cell and thus, the mol­ecules are linked into a mono-periodic hydrogen-bonded ribbon parallel to the *b*-axis. (Fig. 3 ▸).

**Figure 4:**
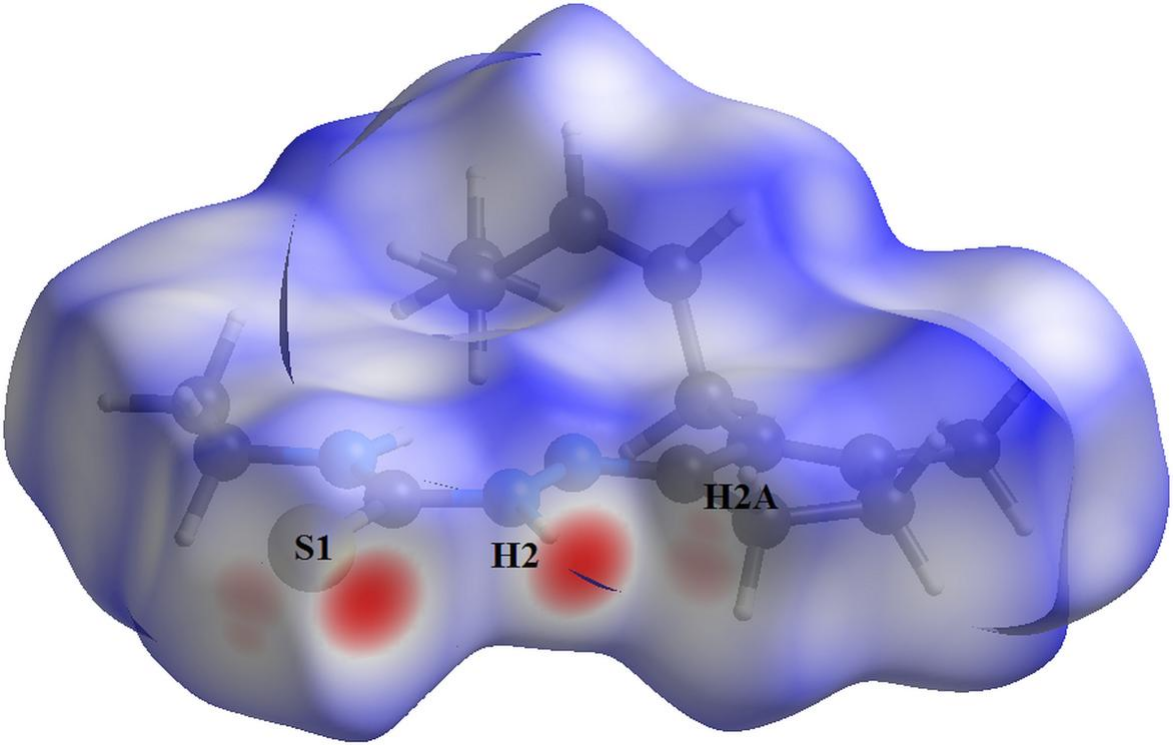
Hirshfeld surface graphical representation (*d*_norm_) for the title compound. The surface is drawn with transparency, the mol­ecules are drawn in *ball and stick* model and the disorder is not shown for clarity. The regions with strongest inter­molecular inter­actions are shown in red.

**Figure 5:**
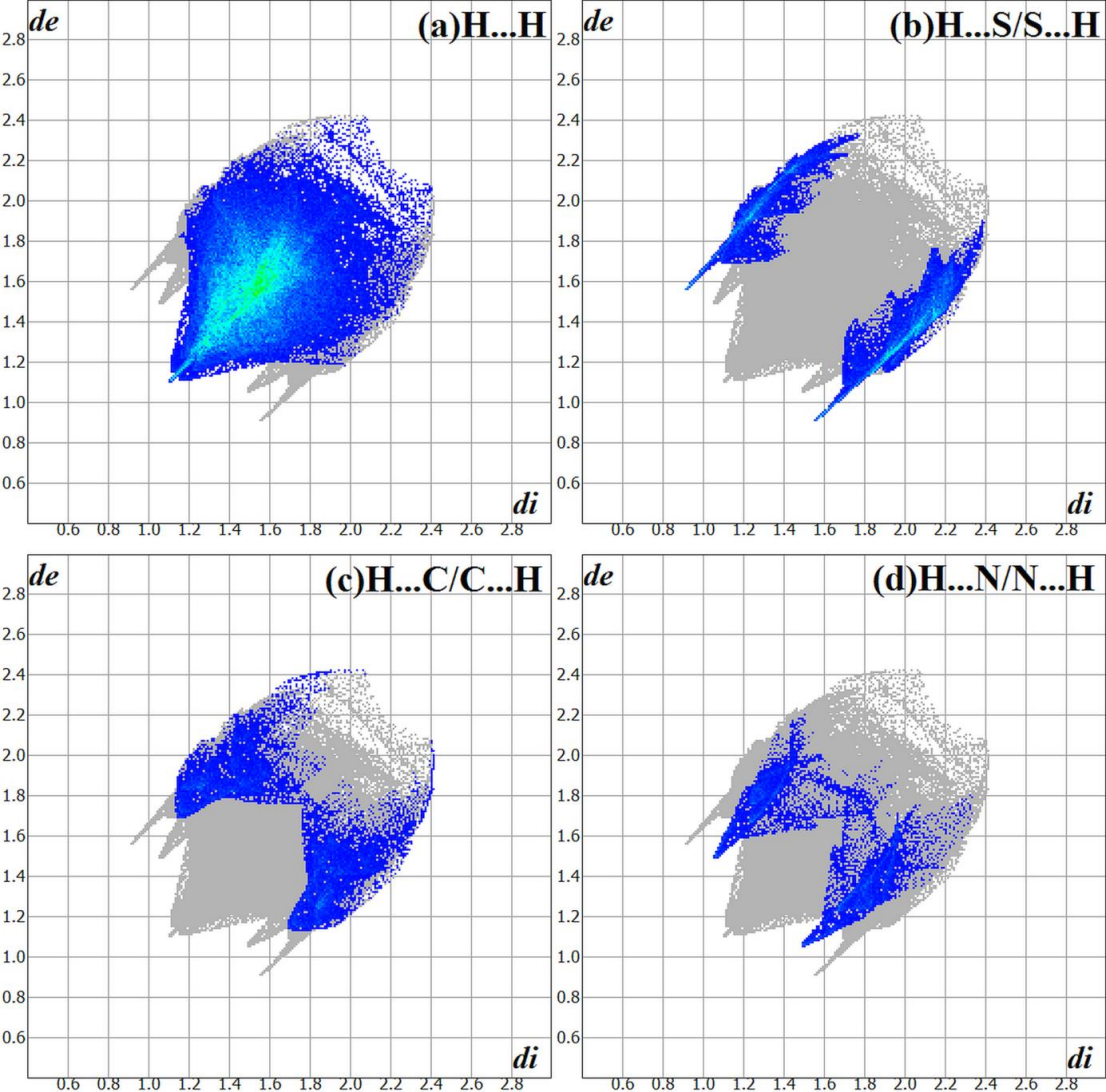
The Hirshfeld surface two-dimensional fingerprint plot (HSFP) for the title compound, showing the contacts in detail (coloured). The major contributions of the inter­actions to the crystal cohesion amount to (*a*) H⋯H (70.7%), (*b*) H⋯S/S⋯H (13.5%), (*c*) H⋯C/C⋯H (8.8%) and (*d*) H⋯N/N⋯H (6.4%). The *d*_i_ (*x-*axis) and the *d*_e_ (*y-*axis) values are the closest inter­nal and external distances from given points on the Hirshfeld surface contacts (in Å). Regarding the disorder, only the atoms with the highest s.o.f. were considered.

The Hirshfeld surface analysis (Hirshfeld, 1977 ▸), the graphical representations and the two-dimensional Hirshfeld surface fingerprint plots (HSFP) were calculated with the *Crystal Explorer* software (Wolff *et al.*, 2012 ▸) and only the atoms with the higher s.o.f. were taken into account. The Hirshfeld surface analysis of the title compound suggests that the most relevant inter­molecular inter­actions for the crystal packing are H⋯H (70.7%), H⋯S/S⋯H (13.5%), H⋯C/C⋯H (8.8%) and H ⋯N/N⋯H (6.4%). The graphical representation of the Hirshfeld surface (*d*_norm_) is given in a figure with transparency and using the *ball-and-stick* model. Locations of the strongest inter­molecular contacts, *i.e*, the regions around the H2, H2*A* and S1 atoms are indicated in red (Fig. 4 ▸). These atoms are those involved in the H⋯S inter­actions shown in previous figures (Figs. 2 ▸ and 3 ▸). The contributions to the crystal cohesion are represented as two-dimensional Hirshfeld surface fingerprint plots (HSFP) with coloured dots (Fig. 5 ▸). The *d*_i_ (*x*-axis) and the *d*_e_ (*y*-axis) values are the closest inter­nal and external distances from given points on the Hirshfeld surface contacts (in Å).

## 4. Database survey

**Figure 6:**
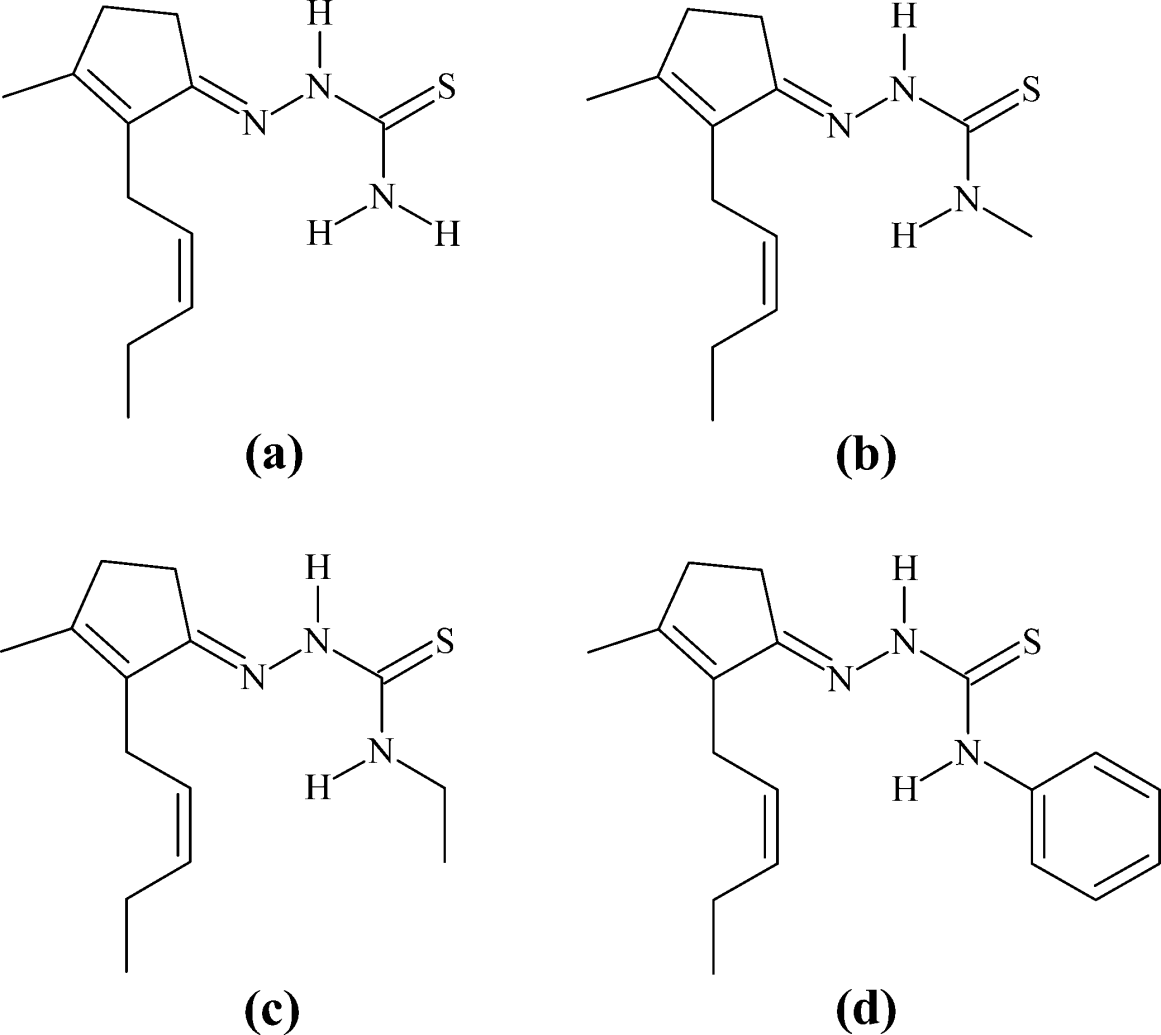
Chemical structure formulae of (*a*) *cis*-jasmone thio­semicarbazone, **JTSC**, (*b*) *cis*-jasmone 4-methyl­thio­semicarbazone, **JMTSC**, (*c*) *cis*-jasmone 4-ethyl­thio­semicarbazone, **JETSC**, and (*d*) *cis*-jasmone 4-phenyl­thio­semicarbazone, **JPTSC**.

**Figure 7:**
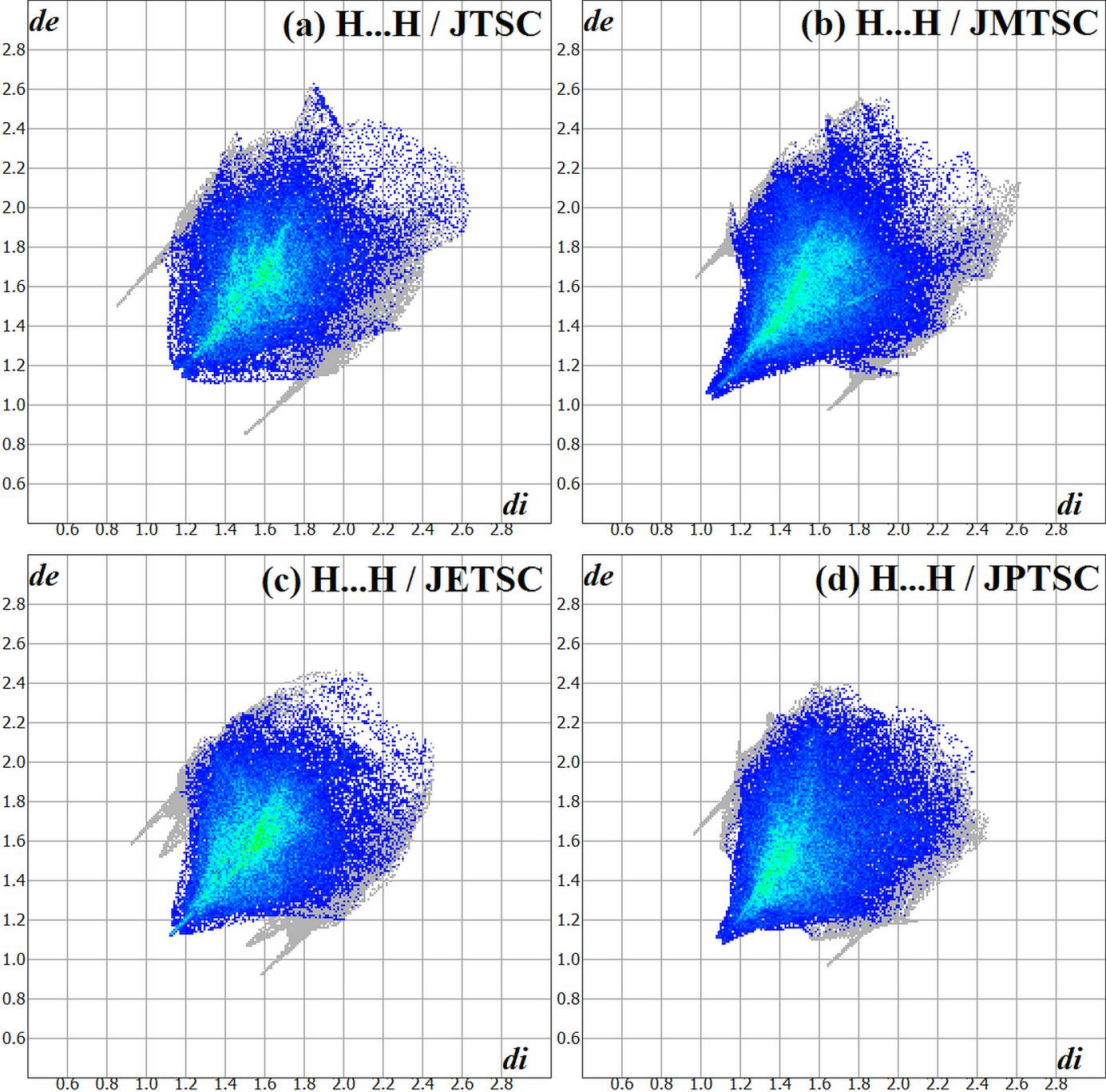
The Hirshfeld surface two-dimensional fingerprint plot (HSFP) signatures for (*a*) **JTSC** (Oliveira *et al.*, 2023*b* ▸), (*b*) **JMTSC** (Oliveira *et al.*, 2024 ▸), (*c*) **JETSC** (this work) and (*d*) **JPTSC** (Oliveira *et al.*, 2023*a* ▸). The major contributions for the crystal cohesion in all structures are the H⋯H inter­molecular inter­actions, which amount to 67.8%, 70.6%, 70.7% and 65.3%, respectively, and are highlighted (coloured). The *d*_i_ (*x-*axis) and the *d*_e_ (*y-*axis) values are the closest inter­nal and external distances from given points on the Hirshfeld surface contacts (in Å). Regarding the disorder, only the atoms with the highest s.o.f. were considered.

**Figure 8:**
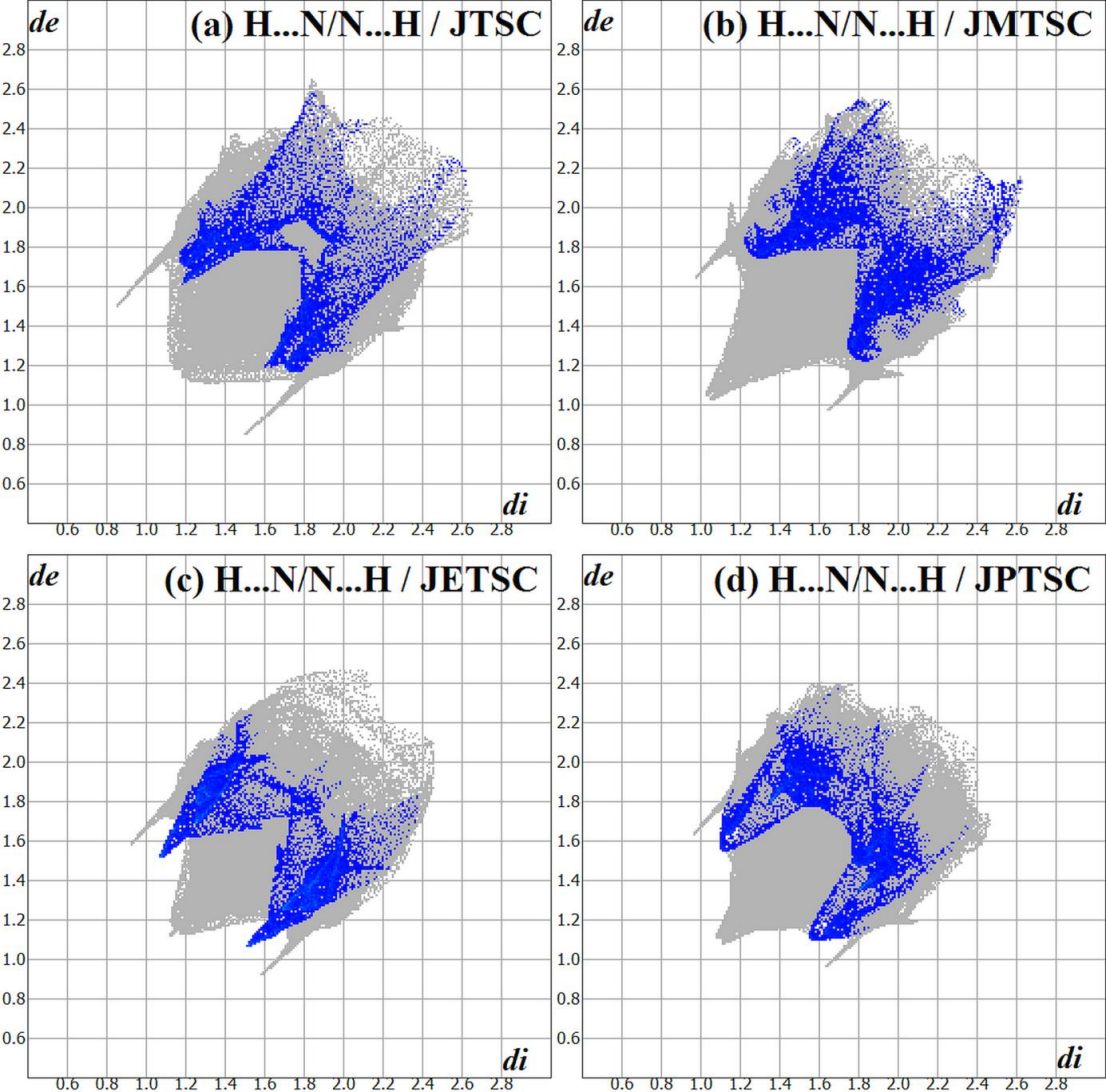
The Hirshfeld surface two-dimensional fingerprint plot (HSFP) signatures for (*a*) **JTSC** (Oliveira *et al.*, 2023*b* ▸), (*b*) **JMTSC** (Oliveira *et al.*, 2024 ▸), (*c*) **JETSC** (this work) and (*d*) **JPTSC** (Oliveira *et al.*, 2023*a* ▸), with the H⋯N/N⋯H contacts highlighted (coloured). The *d*_i_ (*x-*axis) and the *d*_e_ (*y-*axis) values are the closest inter­nal and external distances from given points on the Hirshfeld surface contacts (in Å). Regarding the disorder, only the atoms with the highest s.o.f. were considered.

To the best of our knowledge and from using database tools such as the Cambridge Structural Database (CSD, accessed *via* WebCSD on March 15, 2024; Groom *et al.*, 2016 ▸), there are four crystal structures of *cis*-jasmone thio­semicarbazone derivatives reported in the literature: the α-crystalline modification of *cis*-jasmone thio­semicarbazone, **α-JTSC** (refcode ZAJRUB; Orsoni *et al.*, 2020 ▸), the β-crystalline modification, **β-JTSC** (ZAJRUB01; Oliveira *et al.*, 2023*b* ▸), *cis*-jasmone 4-methyl­thio­semicarbazone, **JMTSC** (JOFYOW; Oliveira *et al.*, 2024 ▸), *cis*-jasmone 4-phenyl­thio­semicarbazone, **JPTSC** (QIVYIH; Oliveira *et al.*, 2023*a* ▸), with *cis*-jasmone 4-ethyl­thio­semicarbazone, **JETSC** (this work) being the fifth. For the Hirshfeld analysis comparison, of the **α-JTSC** and the **β-JTSC** crystalline modifications, only **β-JTSC** was considered and will be designated in the following merely as **JTSC**. Fig. 6 ▸ provides the chemical structures of **JTSC**, **JMTSC**, **JETSC** and **JPTSC**. The Hirshfeld surface fingerprint signatures of the **TSC** derivatives are drawn as two-dimensional plots (HSFP) and the most relevant contribution for the crystal packing, the H⋯H inter­molecular inter­actions, are highlighted (coloured) (Fig. 7 ▸). Their contributions for the crystal cohesion are 67.8% for **JTSC**, 70.6% for **JMTSC**, 70.7% for the title compound, **JETSC**, and 65.3% for **JPTSC**. It might be argued that the methyl and ethyl derivatives show more C—H entities for H⋯H inter­molecular inter­actions in comparison to the non-substituted **JTSC**, and less steric hindrance than the phenyl derivative **JPTSC**. These structural features would explain the higher values of the H⋯H contributions to the crystal packing for **JMTSC** and **JETSC**, and the lower contributions for **JTSC** and **JPTSC**. In addition, the H⋯N/N⋯H contacts, which are important for the supra­molecular arrangement of **JETSC** (this work) are clearly represented in the HSFP signature of the crystal structure and do not appear in the same way in the signature of the related compounds (Fig. 8 ▸). Although the contributions of the H⋯N/N⋯H contacts to the crystal packing for all the jasmone thio­semicarbazone derivatives are very similar in value, within a range of 4.9% to 6.4%, the H⋯N inter­molecular inter­actions are of major importance for the mol­ecular assembly of **JETSC**, as shown in Figs. 2 ▸ and 3 ▸.

**Figure 9:**
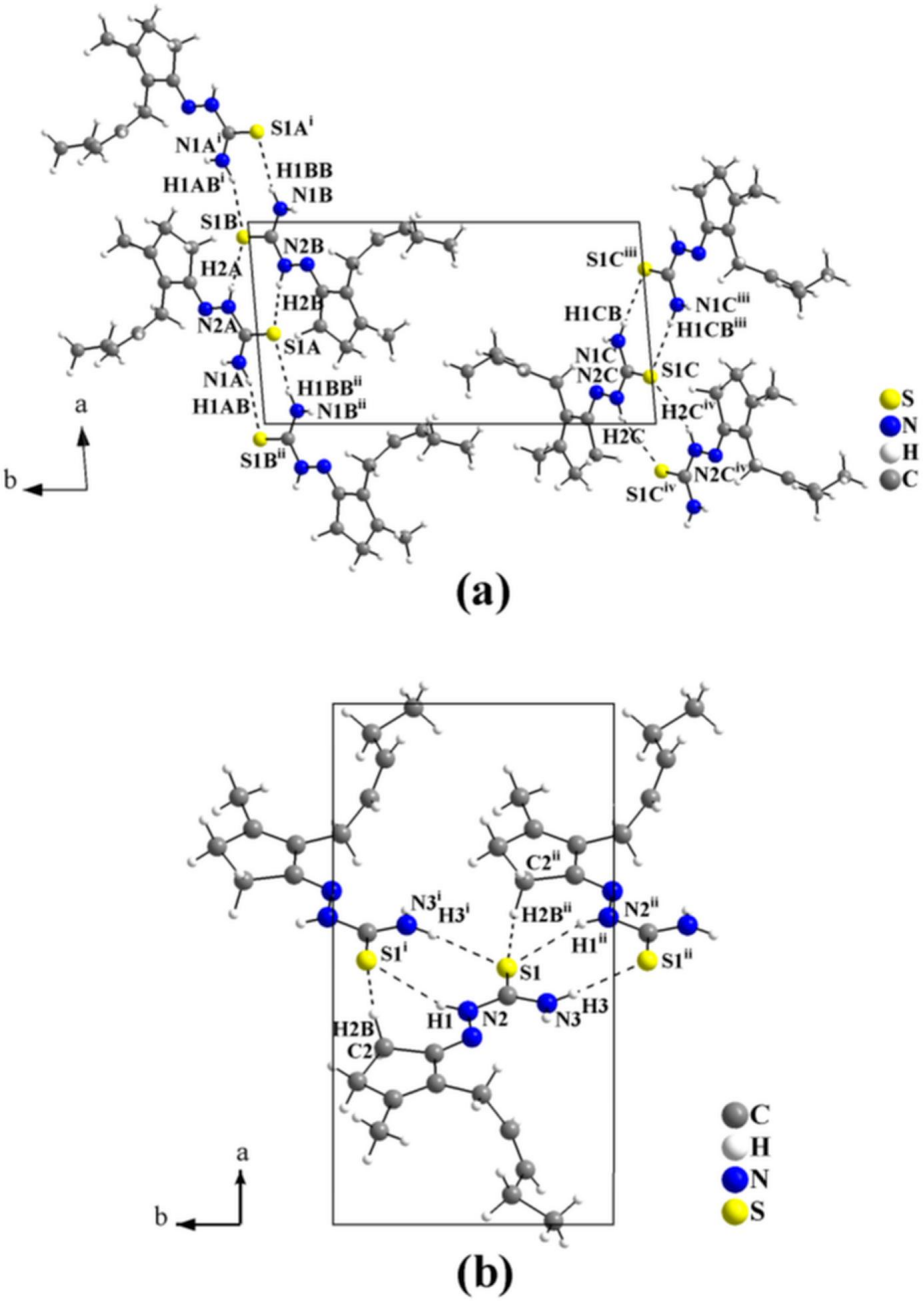
(*a*) Section of the mol­ecular arrangement of **α-JTSC** (Orsoni *et al.*, 2020 ▸). The three crystallographically independent mol­ecules are *A*-, *B*-, and *C*-labelled. They are linked by pairs of N—H⋯S inter­actions, with rings of graph-set motif 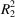 (8), into two independent mono-periodic hydrogen-bonded ribbons along [100]. [Symmetry codes: (i) *x* + 1, *y*, *z*; (ii) *x* − 1, *y*, *z*; (iii) −*x* + 1, −*y*, −*z* + 1; (iv) −*x*, −*y*, −*z* + 1.] (*b*) Section of the mol­ecular arrangement of **β-JTSC** (Oliveira *et al.*, 2023*b* ▸). The mol­ecules are connected by pairs of N—H⋯S and C—H⋯S inter­molecular inter­actions, with rings of graph-set motifs 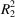 (8) and 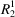 (7), into mono-periodic hydrogen-bonded ribbons along [010]. Disorder is not shown for clarity. [Symmetry codes: (i) −*x* + 1, *y* + 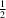 , −*z* + 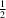 ; (ii) −*x* + 1, *y* − 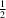 , −*z* + 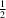 .]

**Figure 10:**
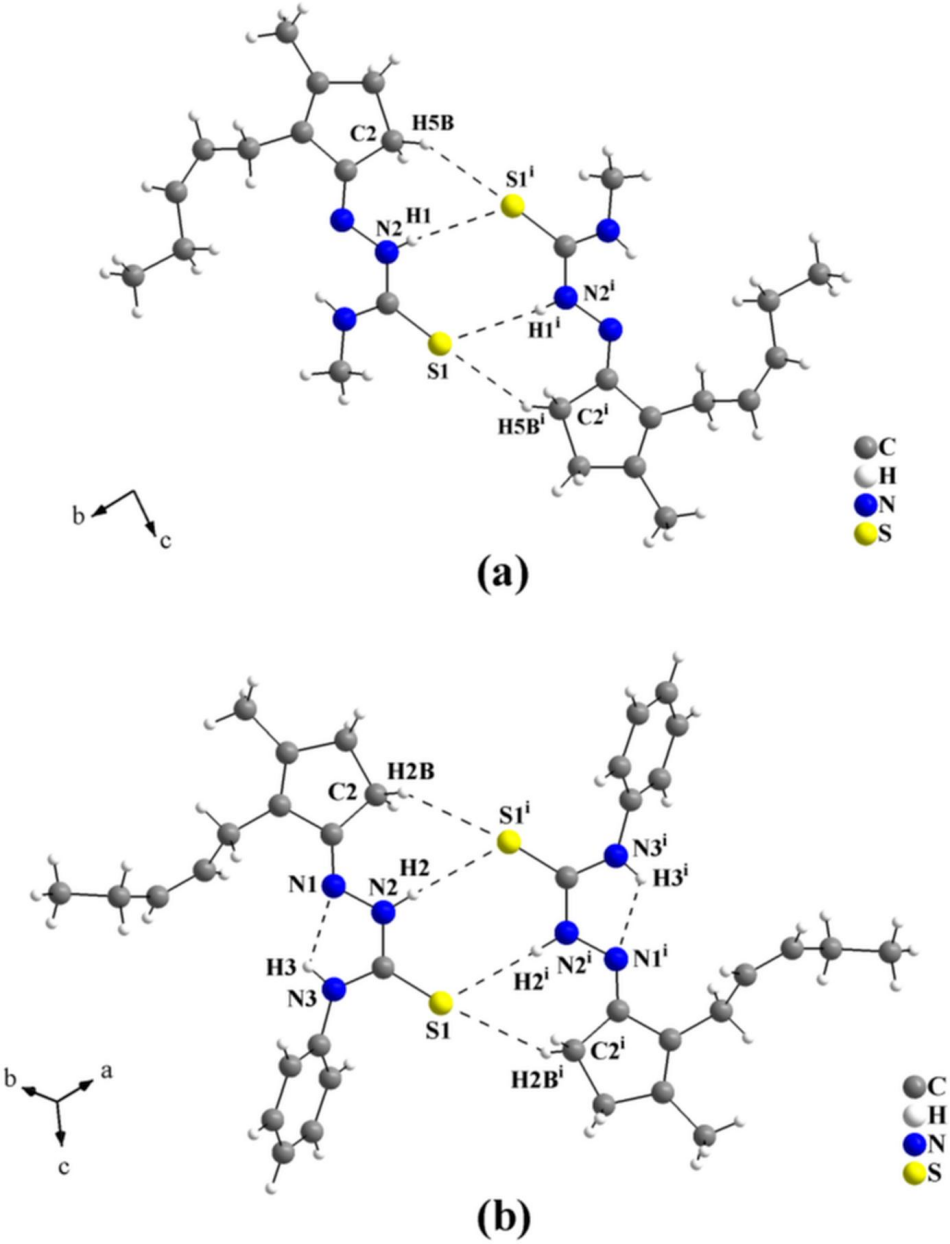
(*a*) Graphical representation of the **JMTSC** dimeric arrangement. The mol­ecules are connected by pairs of N—H⋯S and C—H⋯S inter­molecular inter­actions, with rings of graph-set motifs 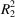 (8) and 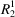 (7), into centrosymmetric dimers. Only the non-disordered **JMTSC-1** dimer is drawn for clarity (Oliveira *et al.*, 2024 ▸). [Symmetry code: (i) −*x*, −*y*, −*z* + 2]. (*b*) Graphical representation of the **JPTSC** dimeric arrangement (Oliveira *et al.*, 2023*a* ▸). The mol­ecules are linked also *via* pairs of N—H⋯S and C—H⋯S inter­molecular inter­actions, with rings of graph-set motifs 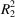 (8) and 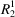 (7), into centrosymmetric dimers. One N—H⋯N intra­molecular inter­action of graph-set *S*(5) is observed. [Symmetry code: (i) −*x* + 1, −*y*, −*z*.]

The influence of the substituent at the terminal N atom on the supra­molecular assembly in the crystal structures of jasmone **TSC** derivatives is shown in Figs. 9 ▸ and 10 ▸. For the non-substituted α- and β-crystalline modifications of *cis*-jasmone thio­semicarbazone, **α-JTSC** (Orsoni *et al.*, 2020 ▸) and **β-JTSC** (Oliveira *et al.*, 2023*b* ▸), the mol­ecules are connected *via* pairs of H⋯S inter­actions into mono-periodic hydrogen-bonded ribbons. The crystal structure of **α-JTSC** shows three crystallographically independent mol­ecules in the asymmetric unit. The mol­ecules are linked by H⋯S inter­actions with graph-set motif 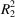 (8) along [100] into two independent one-dimensional hydrogen-bonded polymers (Fig. 9 ▸ *a*). For **β-JTSC**, with one crystallographically independent mol­ecule in the asymmetric unit, the mol­ecules are connected by H⋯S inter­actions with graph-set motifs 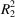 (8) and 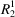 (7) into mono-periodic hydrogen-bonded ribbons along [010] (Fig. 9 ▸ *b*). For the supra­molecular assembly of *cis*-jasmone 4-methyl­thio­semicarbazone, **JMTSC**, (Oliveira *et al.*, 2024 ▸) and of *cis*-jasmone 4-phenyl­thio­semicarbazone, **JPTSC**, (Oliveira *et al.*, 2023*a* ▸), a structural similarity can be observed. In the crystal, the mol­ecules are linked into centrosymmetric dimers by pairs of H⋯S inter­actions, in which rings of graph-set motifs 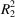 (8) and 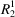 (7) are present. As a result of the steric hindrance of the methyl and phenyl groups, respectively, the dimers are assembled as discrete units and only weak inter­molecular inter­actions, *viz.*, London dispersion forces can be assumed (Fig. 10 ▸ *a*,*b*). The C—H⋯N inter­molecular inter­actions observed in the crystal structure of the title compound, which cause the increase of the supra­molecular dimensionality, are not observed in any of the four crystal structures of closely related mol­ecules mentioned above.

## 5. Synthesis and crystallization

The starting materials are commercially available and were used without further purification. The synthesis of the *cis*-jasmone 4-ethyl­thio­semicarbazone derivative was adapted from previously reported procedures (Freund & Schander, 1902 ▸; Oliveira *et al.*, 2024 ▸ *;* Orsoni *et al.*, 2020 ▸). *cis*-Jasmone was dissolved in ethanol under magnetic stirring at room temperature (8 mmol, 1.3139 g, in 50 mL). A solution of 4-ethyl­thio­semicarbazide in ethanol (8 mmol, 0.9535 g, in 50 mL) was prepared under the same conditions. The solutions were combined, the HCl catalyst was added (1 mL, 1 *M*), and the final mixture was refluxed under magnetic stirring for 8 h. After cooling, the precipitated product was filtered off and washed with cold ethanol. Yield = 0.7431 g (35%). Colourless single crystals suitable for X-ray diffraction were obtained from tetra­hydro­furan by slow evaporation of the solvent at room temperature.

## 6. Refinement

Crystal data, data collection and structure refinement details are summarized in Table 2 ▸. There is one crystallographically independent mol­ecule in the asymmetric unit of the title compound, which shows disorder over the chain of the *cis*-jasmone fragment, *viz.*, the C9 and C10 atoms [Fig. 1 ▸; site-occupancy ratio = 0.911 (5):0.089 (5)]. The H atoms were refined freely, with exception of those bonded to C9*B* and C10*B*. These constrained H atoms were located in a difference-Fourier map, but were positioned with idealized geometry and refined isotropically using a riding model. For the H atoms attached to atom C9*B* with *U*_iso_(H) = 1.2 *U*_eq_(C), the C—H bonds were set to 0.97 Å. For the C10*B* atom, the methyl H atoms were allowed to rotate but not to tip to best fit the experimental electron density, with *U*_iso_(H) = 1.5 *U*_eq_(C), and the C—H bonds were set to 0.96 Å.

## Supplementary Material

Crystal structure: contains datablock(s) I. DOI: 10.1107/S2056989024002913/yz2054sup1.cif

Structure factors: contains datablock(s) I. DOI: 10.1107/S2056989024002913/yz2054Isup2.hkl

Supporting information file. DOI: 10.1107/S2056989024002913/yz2054Isup3.cml

CCDC reference: 2304271

Additional supporting information:  crystallographic information; 3D view; checkCIF report

## Figures and Tables

**Table 1:** Hydrogen-bond geometry (Å, °)

*D*—H⋯*A*	*D*—H	H⋯*A*	*D*⋯*A*	*D*—H⋯*A*
N2—H2⋯S1^i^	0.873 (18)	2.608 (18)	3.4808 (12)	177.6 (15)
N3—H3⋯N1	0.828 (16)	2.187 (15)	2.6008 (15)	111.0 (13)
C2—H2*A*⋯S1^i^	1.003 (16)	2.822 (15)	3.3535 (13)	113.7 (10)
C13—H13*B*⋯N1^ii^	0.966 (15)	2.655 (15)	3.5466 (17)	153.5 (12)

Symmetry codes: (i) 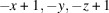 ; (ii) 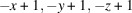 .

**Table 2:** Experimental details

Crystal data	
Chemical formula	C_14_H_23_N_3_S
*M*_r_	265.41
Crystal system, space group	Triclinic, *P* 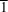
Temperature (K)	123
*a*, *b*, *c* (Å)	7.4584 (2), 7.7429 (3), 13.2461 (3)
α, β, γ (°)	103.025 (2), 98.735 (2), 90.769 (2)
*V* (Å^3^)	735.73 (4)
*Z*	2
Radiation type	Mo *K*α
μ (mm^−1^)	0.21
Crystal size (mm)	0.30 × 0.20 × 0.05

Data collection	
Diffractometer	Enraf–Nonius FR590 Kappa CCD
Absorption correction	Analytical (using the de Meulenaer & Tompa algorithm; Alcock, 1970 ▸)
*T*_min_, *T*_max_	0.944, 0.990
No. of measured, independent and observed [*I* > 2σ(*I*)] reflections	13116, 3325, 2810
*R*_int_	0.047
(sin θ/λ)_max_ (Å^−1^)	0.650

Refinement	
*R*[*F* ^2^ > 2σ(*F* ^2^)], *wR*(*F* ^2^), *S*	0.034, 0.085, 1.02
No. of reflections	3325
No. of parameters	275
H-atom treatment	H atoms treated by a mixture of independent and constrained refinement
Δρ_max_, Δρ_min_ (e Å^−3^)	0.29, −0.21

Computer programs: *COLLECT* (Nonius, 1998 ▸), *HKL*, *DENZO* and *SCALEPACK* (Otwinowski & Minor, 1997 ▸), *SIR92* (Altomare *et al.*, 1994 ▸), *SHELXL2018/3* (Sheldrick, 2015 ▸), *DIAMOND* (Brandenburg, 2006 ▸), *CrystalExplorer* (Wolff *et al.*, 2012 ▸), *WinGX* (Farrugia, 2012 ▸), *publCIF* (Westrip, 2010 ▸) and *enCIFer* (Allen *et al.*, 2004 ▸).

## supplementary crystallographic information

### Crystal data

**Table:**

C_14_H_23_N_3_S	*Z* = 2
*M**_r_* = 265.41	*F*(000) = 288
Triclinic, *P*1	*D*_x_ = 1.198 Mg m^−^^3^
*a* = 7.4584 (2) Å	Mo *K*α radiation, λ = 0.71073 Å
*b* = 7.7429 (3) Å	Cell parameters from 27549 reflections
*c* = 13.2461 (3) Å	θ = 2.9–27.5°
α = 103.025 (2)°	µ = 0.21 mm^−^^1^
β = 98.735 (2)°	*T* = 123 K
γ = 90.769 (2)°	Fragment, colourless
*V* = 735.73 (4) Å^3^	0.30 × 0.20 × 0.05 mm

### Data collection

**Table:**

Enraf–Nonius FR590 Kappa CCD diffractometer	3325 independent reflections
Radiation source: sealed X-ray tube, Enraf Nonius FR590	2810 reflections with *I* > 2σ(*I*)
Horizontally mounted graphite crystal monochromator	*R*_int_ = 0.047
Detector resolution: 9 pixels mm^-1^	θ_max_ = 27.5°, θ_min_ = 3.0°
CCD rotation images, thick slices, κ–goniostat scans	*h* = −9→9
Absorption correction: analytical (using the de Meulenaer & Tompa algorithm; Alcock, 1970)	*k* = −10→10
*T*_min_ = 0.944, *T*_max_ = 0.990	*l* = −17→17
13116 measured reflections	

### Refinement

**Table:**

Refinement on *F*^2^	Primary atom site location: structure-invariant direct methods
Least-squares matrix: full	Secondary atom site location: difference Fourier map
*R*[*F*^2^ > 2σ(*F*^2^)] = 0.034	Hydrogen site location: mixed
*wR*(*F*^2^) = 0.085	H atoms treated by a mixture of independent and constrained refinement
*S* = 1.02	*w* = 1/[σ^2^(*F*_o_^2^) + (0.0336*P*)^2^ + 0.2782*P*] where *P* = (*F*_o_^2^ + 2*F*_c_^2^)/3
3325 reflections	(Δ/σ)_max_ < 0.001
275 parameters	Δρ_max_ = 0.28 e Å^−^^3^
0 restraints	Δρ_min_ = −0.21 e Å^−^^3^

### Special details

**Table:**

Geometry. All esds (except the esd in the dihedral angle between two l.s. planes) are estimated using the full covariance matrix. The cell esds are taken into account individually in the estimation of esds in distances, angles and torsion angles; correlations between esds in cell parameters are only used when they are defined by crystal symmetry. An approximate (isotropic) treatment of cell esds is used for estimating esds involving l.s. planes.

### Fractional atomic coordinates and isotropic or equivalent isotropic displacement parameters (Å^2^)

**Table:**

	*x*	*y*	*z*	*U*_iso_*/*U*_eq_	Occ. (<1)
C1	0.87223 (16)	0.34727 (16)	0.71554 (9)	0.0198 (3)	
C2	0.95725 (17)	0.17051 (17)	0.68820 (10)	0.0224 (3)	
C3	1.14804 (18)	0.20144 (18)	0.75527 (11)	0.0243 (3)	
C4	1.16282 (17)	0.39689 (17)	0.80623 (10)	0.0217 (3)	
C5	1.00677 (16)	0.47688 (17)	0.78541 (9)	0.0207 (3)	
C6	0.96603 (18)	0.66839 (18)	0.82233 (11)	0.0246 (3)	
C7	0.8584 (2)	0.69971 (19)	0.91194 (11)	0.0304 (3)	
C8	0.7226 (2)	0.8050 (2)	0.92470 (12)	0.0328 (3)	
C9A	0.6346 (2)	0.9092 (3)	0.84930 (14)	0.0342 (5)	0.911 (5)
C10A	0.6880 (4)	1.1062 (3)	0.8879 (2)	0.0465 (6)	0.911 (5)
H9A	0.500 (3)	0.894 (3)	0.8412 (16)	0.050 (6)*	0.911 (5)
H9B	0.666 (2)	0.859 (2)	0.7779 (15)	0.036 (5)*	0.911 (5)
H10A	0.823 (3)	1.124 (3)	0.8960 (16)	0.048 (6)*	0.911 (5)
H10B	0.625 (3)	1.173 (3)	0.837 (2)	0.065 (7)*	0.911 (5)
H10C	0.655 (3)	1.149 (3)	0.959 (2)	0.063 (7)*	0.911 (5)
C9B	0.778 (3)	1.006 (2)	0.9103 (13)	0.033 (5)	0.089 (5)
H9C	0.794526	1.092924	0.976673	0.039*	0.089 (5)
H9D	0.886258	1.005830	0.878229	0.039*	0.089 (5)
C10B	0.614 (3)	1.036 (3)	0.839 (2)	0.042 (6)	0.089 (5)
H10D	0.595042	0.941005	0.777614	0.064*	0.089 (5)
H10E	0.630332	1.146535	0.819865	0.064*	0.089 (5)
H10F	0.510340	1.040027	0.874748	0.064*	0.089 (5)
C11	1.33750 (18)	0.4800 (2)	0.87064 (12)	0.0284 (3)	
C12	0.43231 (16)	0.31557 (17)	0.57148 (10)	0.0203 (3)	
C13	0.22713 (17)	0.56201 (18)	0.56951 (11)	0.0226 (3)	
C14	0.2314 (2)	0.75737 (19)	0.62214 (12)	0.0279 (3)	
H2	0.627 (2)	0.157 (2)	0.5899 (13)	0.037 (5)*	
H3	0.477 (2)	0.548 (2)	0.6476 (12)	0.026 (4)*	
H2A	0.883 (2)	0.072 (2)	0.7024 (12)	0.029 (4)*	
H2B	0.967 (2)	0.139 (2)	0.6138 (13)	0.028 (4)*	
H3A	1.243 (2)	0.167 (2)	0.7122 (12)	0.030 (4)*	
H3B	1.163 (2)	0.132 (2)	0.8094 (12)	0.029 (4)*	
H6A	0.900 (2)	0.712 (2)	0.7621 (12)	0.028 (4)*	
H6B	1.081 (2)	0.739 (2)	0.8457 (12)	0.033 (4)*	
H7	0.899 (3)	0.636 (3)	0.9680 (15)	0.054 (5)*	
H8	0.670 (2)	0.817 (2)	0.9901 (14)	0.040 (5)*	
H11A	1.438 (3)	0.449 (2)	0.8318 (14)	0.045 (5)*	
H11B	1.335 (2)	0.609 (3)	0.8935 (14)	0.046 (5)*	
H11C	1.366 (3)	0.434 (3)	0.9314 (15)	0.051 (5)*	
H13A	0.127 (2)	0.4975 (19)	0.5857 (11)	0.021 (3)*	
H13B	0.211 (2)	0.5436 (19)	0.4940 (12)	0.024 (4)*	
H14A	0.244 (2)	0.775 (2)	0.6981 (13)	0.032 (4)*	
H14B	0.332 (2)	0.821 (2)	0.6068 (13)	0.035 (4)*	
H14C	0.118 (2)	0.805 (2)	0.5956 (13)	0.038 (4)*	
N1	0.71169 (14)	0.39345 (14)	0.68391 (8)	0.0214 (2)	
N2	0.59457 (14)	0.26386 (15)	0.61441 (9)	0.0222 (2)	
N3	0.39593 (14)	0.48485 (15)	0.60530 (9)	0.0217 (2)	
S1	0.28777 (4)	0.16924 (4)	0.48084 (3)	0.02554 (11)	

### Atomic displacement parameters (Å^2^)

**Table:**

	*U*^11^	*U*^22^	*U*^33^	*U*^12^	*U*^13^	*U*^23^
C1	0.0176 (6)	0.0227 (6)	0.0190 (6)	−0.0003 (5)	0.0017 (5)	0.0055 (5)
C2	0.0184 (6)	0.0233 (6)	0.0231 (7)	−0.0006 (5)	−0.0009 (5)	0.0031 (5)
C3	0.0187 (6)	0.0257 (7)	0.0261 (7)	0.0019 (5)	−0.0009 (5)	0.0039 (5)
C4	0.0192 (6)	0.0255 (6)	0.0196 (6)	−0.0020 (5)	0.0005 (5)	0.0052 (5)
C5	0.0199 (6)	0.0226 (6)	0.0189 (6)	−0.0021 (5)	0.0019 (5)	0.0047 (5)
C6	0.0225 (6)	0.0222 (6)	0.0277 (7)	−0.0017 (5)	0.0010 (5)	0.0048 (5)
C7	0.0354 (8)	0.0288 (7)	0.0275 (7)	0.0040 (6)	0.0050 (6)	0.0075 (6)
C8	0.0354 (8)	0.0311 (8)	0.0325 (8)	0.0031 (6)	0.0095 (6)	0.0058 (6)
C9A	0.0276 (9)	0.0372 (12)	0.0351 (10)	0.0066 (7)	0.0011 (7)	0.0050 (8)
C10A	0.0618 (16)	0.0311 (12)	0.0514 (15)	0.0110 (11)	0.0225 (12)	0.0099 (10)
C9B	0.046 (10)	0.017 (8)	0.032 (9)	0.012 (7)	0.010 (7)	−0.003 (6)
C10B	0.048 (13)	0.037 (14)	0.054 (14)	0.011 (11)	0.043 (12)	0.010 (11)
C11	0.0203 (7)	0.0318 (8)	0.0291 (8)	−0.0025 (6)	−0.0040 (6)	0.0041 (6)
C12	0.0163 (6)	0.0234 (6)	0.0217 (6)	−0.0009 (5)	0.0021 (5)	0.0068 (5)
C13	0.0173 (6)	0.0263 (7)	0.0236 (7)	0.0016 (5)	−0.0003 (5)	0.0070 (5)
C14	0.0239 (7)	0.0276 (7)	0.0306 (8)	0.0051 (6)	0.0024 (6)	0.0043 (6)
N1	0.0184 (5)	0.0227 (5)	0.0212 (5)	−0.0025 (4)	−0.0009 (4)	0.0042 (4)
N2	0.0169 (5)	0.0204 (6)	0.0258 (6)	−0.0002 (4)	−0.0027 (4)	0.0023 (4)
N3	0.0161 (5)	0.0222 (5)	0.0234 (6)	−0.0003 (4)	−0.0028 (4)	0.0021 (4)
S1	0.01824 (16)	0.02307 (18)	0.03029 (19)	−0.00124 (12)	−0.00422 (12)	0.00120 (13)

### Geometric parameters (Å, º)

**Table:**

C1—N1	1.2899 (16)	C10A—H10B	1.01 (3)
C1—C5	1.4639 (17)	C10A—H10C	0.99 (2)
C1—C2	1.5086 (18)	C9B—C10B	1.48 (3)
C2—C3	1.5436 (17)	C9B—H9C	0.9700
C2—H2A	1.003 (16)	C9B—H9D	0.9700
C2—H2B	0.974 (16)	C10B—H10D	0.9600
C3—C4	1.5068 (18)	C10B—H10E	0.9600
C3—H3A	0.977 (16)	C10B—H10F	0.9600
C3—H3B	0.980 (16)	C11—H11A	0.973 (19)
C4—C5	1.3465 (17)	C11—H11B	0.98 (2)
C4—C11	1.4939 (18)	C11—H11C	0.95 (2)
C5—C6	1.5035 (18)	C12—N3	1.3319 (17)
C6—C7	1.509 (2)	C12—N2	1.3620 (16)
C6—H6A	0.998 (16)	C12—S1	1.6877 (13)
C6—H6B	0.980 (17)	C13—N3	1.4606 (16)
C7—C8	1.316 (2)	C13—C14	1.5139 (19)
C7—H7	0.99 (2)	C13—H13A	0.971 (15)
C8—C9A	1.502 (2)	C13—H13B	0.966 (15)
C8—C9B	1.661 (17)	C14—H14A	0.975 (17)
C8—H8	0.992 (18)	C14—H14B	0.963 (17)
C9A—C10A	1.522 (3)	C14—H14C	0.973 (18)
C9A—H9A	0.99 (2)	N1—N2	1.3904 (15)
C9A—H9B	1.00 (2)	N2—H2	0.873 (18)
C10A—H10A	1.00 (2)	N3—H3	0.828 (16)
			
N1—C1—C5	120.80 (11)	C9A—C10A—H10C	109.0 (14)
N1—C1—C2	129.87 (11)	H10A—C10A—H10C	106.4 (18)
C5—C1—C2	109.28 (10)	H10B—C10A—H10C	111 (2)
C1—C2—C3	103.99 (10)	C10B—C9B—C8	100.0 (14)
C1—C2—H2A	111.9 (9)	C10B—C9B—H9C	111.8
C3—C2—H2A	113.2 (9)	C8—C9B—H9C	111.8
C1—C2—H2B	110.7 (9)	C10B—C9B—H9D	111.8
C3—C2—H2B	110.4 (9)	C8—C9B—H9D	111.8
H2A—C2—H2B	106.7 (12)	H9C—C9B—H9D	109.5
C4—C3—C2	104.68 (10)	C9B—C10B—H10D	109.5
C4—C3—H3A	112.1 (9)	C9B—C10B—H10E	109.5
C2—C3—H3A	111.3 (9)	H10D—C10B—H10E	109.5
C4—C3—H3B	109.8 (9)	C9B—C10B—H10F	109.5
C2—C3—H3B	112.2 (9)	H10D—C10B—H10F	109.5
H3A—C3—H3B	106.8 (13)	H10E—C10B—H10F	109.5
C5—C4—C11	127.60 (12)	C4—C11—H11A	110.2 (11)
C5—C4—C3	112.28 (11)	C4—C11—H11B	112.8 (11)
C11—C4—C3	120.11 (11)	H11A—C11—H11B	110.0 (15)
C4—C5—C1	109.42 (11)	C4—C11—H11C	110.6 (12)
C4—C5—C6	128.51 (12)	H11A—C11—H11C	105.0 (15)
C1—C5—C6	122.06 (11)	H11B—C11—H11C	107.9 (15)
C5—C6—C7	113.14 (11)	N3—C12—N2	116.44 (11)
C5—C6—H6A	109.4 (9)	N3—C12—S1	122.95 (10)
C7—C6—H6A	110.4 (9)	N2—C12—S1	120.61 (10)
C5—C6—H6B	108.9 (9)	N3—C13—C14	110.15 (11)
C7—C6—H6B	108.3 (9)	N3—C13—H13A	108.2 (8)
H6A—C6—H6B	106.6 (13)	C14—C13—H13A	111.2 (9)
C8—C7—C6	127.15 (14)	N3—C13—H13B	109.0 (9)
C8—C7—H7	117.6 (11)	C14—C13—H13B	111.4 (9)
C6—C7—H7	115.2 (11)	H13A—C13—H13B	106.7 (12)
C7—C8—C9A	126.93 (15)	C13—C14—H14A	111.1 (9)
C7—C8—C9B	110.7 (6)	C13—C14—H14B	111.1 (10)
C7—C8—H8	117.8 (10)	H14A—C14—H14B	107.7 (14)
C9A—C8—H8	115.3 (10)	C13—C14—H14C	108.0 (10)
C9B—C8—H8	109.2 (11)	H14A—C14—H14C	109.8 (14)
C8—C9A—C10A	111.28 (16)	H14B—C14—H14C	109.1 (14)
C8—C9A—H9A	110.1 (12)	C1—N1—N2	117.11 (11)
C10A—C9A—H9A	108.3 (13)	C12—N2—N1	117.59 (11)
C8—C9A—H9B	109.3 (11)	C12—N2—H2	118.7 (11)
C10A—C9A—H9B	112.1 (11)	N1—N2—H2	122.9 (11)
H9A—C9A—H9B	105.6 (16)	C12—N3—C13	123.81 (11)
C9A—C10A—H10A	109.6 (13)	C12—N3—H3	116.1 (11)
C9A—C10A—H10B	109.5 (16)	C13—N3—H3	120.0 (11)
H10A—C10A—H10B	111 (2)		
			
N1—C1—C2—C3	−177.86 (13)	C1—C5—C6—C7	79.84 (15)
C5—C1—C2—C3	4.82 (14)	C5—C6—C7—C8	−138.74 (16)
C1—C2—C3—C4	−5.70 (13)	C6—C7—C8—C9A	4.5 (3)
C2—C3—C4—C5	5.04 (15)	C6—C7—C8—C9B	−50.1 (6)
C2—C3—C4—C11	−174.65 (12)	C7—C8—C9A—C10A	−106.5 (2)
C11—C4—C5—C1	177.60 (13)	C7—C8—C9B—C10B	132.7 (10)
C3—C4—C5—C1	−2.06 (15)	C5—C1—N1—N2	177.52 (10)
C11—C4—C5—C6	−1.1 (2)	C2—C1—N1—N2	0.47 (19)
C3—C4—C5—C6	179.29 (12)	N3—C12—N2—N1	−4.03 (17)
N1—C1—C5—C4	−179.51 (11)	S1—C12—N2—N1	176.25 (9)
C2—C1—C5—C4	−1.91 (14)	C1—N1—N2—C12	−172.87 (11)
N1—C1—C5—C6	−0.76 (18)	N2—C12—N3—C13	−178.66 (11)
C2—C1—C5—C6	176.84 (11)	S1—C12—N3—C13	1.04 (18)
C4—C5—C6—C7	−101.67 (16)	C14—C13—N3—C12	−179.07 (12)

### Hydrogen-bond geometry (Å, º)

**Table:**

*D*—H···*A*	*D*—H	H···*A*	*D*···*A*	*D*—H···*A*
N2—H2···S1^i^	0.873 (18)	2.608 (18)	3.4808 (12)	177.6 (15)
N3—H3···N1	0.828 (16)	2.187 (15)	2.6008 (15)	111.0 (13)
C2—H2*A*···S1^i^	1.003 (16)	2.822 (15)	3.3535 (13)	113.7 (10)
C13—H13*B*···N1^ii^	0.966 (15)	2.655 (15)	3.5466 (17)	153.5 (12)

Symmetry codes: (i) −*x*+1, −*y*, −*z*+1; (ii) −*x*+1, −*y*+1, −*z*+1.
